# ‘Getting addicted to it and losing a lot of money… it’s just like a hole.’ A grounded theory model of how social determinants shape adolescents’ choices to not gamble

**DOI:** 10.1186/s12889-024-18286-3

**Published:** 2024-05-09

**Authors:** Nerilee Hing, Hannah Thorne, Lisa Lole, Kerry Sproston, Nicole Hodge, Matthew Rockloff

**Affiliations:** 1 Experimental Gambling Research Laboratory, School of Health, Medical and Applied Sciences, CQUniversity, University Drive, 4670 Bundaberg, QLD Australia; 2 Experimental Gambling Research Laboratory, School of Health, Medical and Applied Sciences, CQUniversity, Adelaide, South Australia Australia; 3 DBM Consultants, Melbourne, VIC Australia

**Keywords:** Youth, Adolescents, Gambling, Protective factors, Social determinants, Qualitative methods, Grounded theory

## Abstract

**Background:**

Gambling abstinence when underage lowers the risk of harmful gambling in later life. However, little research has examined *why* many young people refrain from gambling, even though this knowledge can inform protective strategies and lower risk factors to reduce underage gambling and subsequent harm. This study draws on the lived experience of adolescent non-gamblers to explore how social determinants while growing up have shaped their reasons and choices to not gamble.

**Methods:**

Fourteen Australian non-gamblers, aged 12–17 years, participated in an in-depth individual interview (4 girls, 3 boys) or online community (4 girls, 3 boys). Questions in each condition differed, but both explored participants’ gambling-related experiences while growing up, including exposure, attitudes and behaviours of parents and peers, advertising, simulated gambling and motivations for not gambling. The analysis used adaptive grounded theory methods.

**Results:**

The grounded theory model identifies several reasons for not gambling, including not being interested, being below the legal gambling age, discouragement from parent and peers, concern about gambling addiction and harm, not wanting to risk money on a low chance of winning, and moral objections. These reasons were underpinned by several social determinants, including individual, parental, peer and environmental factors that can interact to deter young people from underage gambling. Key protective factors were parental role modelling and guidance, friendship groups who avoided gambling, critical thinking, rational gambling beliefs, financial literacy and having other hobbies and interests.

**Conclusions:**

Choices to not gamble emanated from multiple layers of influence, implying that multi-layered interventions, aligned with a public health response, are needed to deter underage gambling. At the environmental level, better age-gating for monetary and simulated gambling, countering cultural pressures, and less exposure to promotional gambling messages, may assist young people to resist these influences. Interventions that support parents to provide appropriate role modelling and guidance for their children are also important. Youth education could include cautionary tales from people with lived experience of gambling harm, and education to increase young people’s financial literacy, ability to recognise marketing tactics, awareness of the risks and harms of gambling, and how to resist peer and other normalising gambling influences.

**Supplementary Information:**

The online version contains supplementary material available at 10.1186/s12889-024-18286-3.

## Background

Most research into gambling amongst adolescents has focused on the prevalence and predictors of harmful gambling [1, 2]. Since early engagement in gambling is a risk factor for gambling problems in adulthood [3, 4], studies have also examined the reasons that adolescents participate in gambling when underage [5, 6]. However, little attention has focused on understanding why many young people *refrain* from gambling. Approximately 50–70% of adolescents report no past-year gambling [7, 8], even though underage access to many gambling products is reportedly easy [9]. Understanding why these adolescents choose to refrain from gambling can inform protective strategies against underage gambling and subsequent gambling harm.

Numerous theoretical models identify the key types of influences on youth developmental outcomes [10, 11], health outcomes [12, 13], and the development of gambling behaviours and subsequent harms [14–17]. These models all recognise that these behaviours and outcomes are influenced by complex interactions between multiple factors (e.g., individual attributes; physical, cultural and social circumstances) and at multiple levels (e.g., individuals, relationships, organisations, society). This recognition that multiple and multi-level factors impact on health behaviours and outcomes can inform an understanding of how various influences interact to shape young people’s decisions to refrain from gambling.

### Young people’s self-reported reasons for not gambling

To our knowledge, only two survey studies have examined reasons for not gambling amongst young people. Rash and McGrath [18] conducted a content analysis of responses to an open-ended survey question asked of 196 Canadian undergraduates (mean age = 21.2 years, *SD* = 3.7) who reported no past-year gambling. They were asked to ‘think about what motivates you to NOT gamble and briefly list the top three reasons in rank order.’ The most common motive was financial reasons and risk aversion (33.1%), followed by disinterest/other priorities (21.1%), personal and religious objections (12.2%), addiction concerns (9.6%), influence of others’ values (9.1%), awareness of the odds (8.9%), lack of access, opportunity or skill (2.1%) and emotional distress (1.7%).

Another study focused specifically on young people under the legal gambling age [7]. It surveyed a weighted sample of 2559 students aged 11–16 years in England, Scotland and Wales. Those who reported no past-year gambling were asked: ‘You said that you have never gambled or never spent your own money on gambling. Why is that?’ and were provided with multiple response options. The most endorsed reasons were lack of interest in gambling (39%), because it is illegal or they thought they were too young (37%), not wanting to play with real money/rather play with free games (25%), not being allowed to gamble by their parents (24%), and because it may lead to future problems (22%). Less common reasons were expecting to lose more than they will win (21%), because they ‘don’t agree with gambling and/or it is not right’ (21%), thinking they were unlikely to win money (19%), not knowing enough about gambling games (11%) and religious objections (10%). Girls tended to report less interest in gambling, while boys were more likely to cite that gambling may lead to future problems. Younger participants were more likely to endorse that they did not agree with gambling and that their parents do not allow them to gamble. These findings align with observations that adolescent non-gamblers tend to be female and younger, compared to adolescent gamblers [19–21].

### Social determinants of adolescent non-gambling

Social determinants of health are the non-medical factors that influence health outcomes [22]. Several social determinants may directly and indirectly shape the reasons for not gambling that many young people report, although this linkage has not previously been examined. Nonetheless, studies that compare non-gamblers to gamblers amongst adolescents provide some insights into social factors associated with non-gambling.

In a survey of 506 students from six schools in South Australia (mean age = 16.5, *SD* = 0.77 years), non-gamblers rated gambling as more unprofitable, compared to gamblers, and were significantly less likely to have family or friends who approved of gambling or who gambled a lot [23]. In another Australian study of students aged 12–17 years in Queensland and Victoria (*N* = 6377), those who had not gambled in the past month were significantly more likely than past-month gamblers to report having less spending money available, lower alcohol consumption, less exposure to gambling advertisements, and fewer peers or family members who had recently gambled [20]. Also in Australia, unique predictors of past-year non-gambling identified in two non-probability samples of youth aged 12–17 years (*N* = 826, *N* = 843) were parental disapproval of gambling, not gambling with their parents while growing up, not having friends who gambled, and avoidance of simulated gambling [8].

In New Zealand, Rossen [21] surveyed students from 12 secondary schools (*N* = 2005; mean age = 15.2 years, *SD* = 1.45). Compared to gamblers, non-gamblers tended to have lower rates of internet and computer game usage, alcohol usage, and recall of seeing gambling advertising. They were also less likely to have family members or friends who gambled or had a gambling problem. Further, less liberal attitudes to gambling, lower perceived ease of access to gambling, and lower perceived role of skill in gambling were associated with non-gambling status. Non-gambling was also associated with being required to contribute to household chores, higher importance of spiritual beliefs, higher parental attachment, trust and communication, and lower maternal, paternal and peer alienation.

In the US, a survey of 15,865 eighth-graders in Oregon (mean age = 13.7 years, *SD* = 0.50) focused on health behaviours, including gambling during the previous three months [24]. Good personal safety habits, non-involvement in antisocial behaviour, and strong personal health beliefs predicted non-gambling in both girls and boys. Amongst girls, non-gamblers were also more likely than gamblers to report less screen time on school nights, no tobacco use, and to speak English at home. Amongst boys, living in neighbourhoods with strong social control and non-Hispanic ethnicity also predicted non-gambling. Also in North America, a study of students aged 13–19 years in Canada (*N* = 10,035) found that non-gamblers were less likely to engage in simulated gambling, compared to those who gambled [19].

In summary, two studies have examined qualitative self-reported reasons given by young people for not gambling, while quantitative research identifies social factors that differ between adolescent non-gamblers and gamblers. However, a detailed exploration linking reasons for not gambling with social factors is lacking. This study therefore aims to draw on the lived experience of adolescent non-gamblers to explore how social determinants can shape their reasons and choices to not gamble as they grow up.

## Methods

We use a grounded theory methodology in this study, which is appropriate when a research topic lacks a theoretical foundation. This approach allows us to expand upon previous reasons that adolescents report for not gambling to also identify underlying social determinants and processes. The study was approved by our institutional ethics committee (number 23,445).

### Recruitment

Participants were adolescents aged 12–17 years who lived in NSW and provided their own and their legal guardian’s informed consent. Due to ethical concerns surrounding anonymity, confidentiality, and minimising legal risk to underage participants, detailed information on participants was not collected. Sampling ensured reasonably even representation from younger (12–14 years) and older (15–17 years) ages, boys and girls, as well as regional and metropolitan locations (Tables 1 and 2).

**Table 1:** Demographic details for participants in the individual interview condition

Participant #	Gender	Age bracket	Location
1	M	15–17	Regional
2	M	12–14	Regional
3	M	12–14	Metro
4	F	15–17	Metro
5	F	15–17	Regional
6	F	12–14	Regional
7	F	12–14	Metro

**Table 2:** Demographic details for participants in the online community condition

Participant #	Gender	Age bracket	Location
8	M	12–14	Metro
9	M	15–17	Metro
10	M	15–17	Regional
11	F	12–14	Metro
12	F	12–14	Regional
13	F	15–17	Metro
14	F	15–17	Regional

Parents/guardians in the recruitment agency’s database were the initial point of contact to recruit the adolescents to participate in either an interview or online community. The funding agency requested these options be offered, based on the rationale that the strengths and weaknesses of each method would complement each other. The parents were contacted via email with an information sheet and invited to ask their adolescent to complete a brief online recruitment screener, which included questions confirming no past-year adolescent gambling, basic demographics, and confirmation of their and their parent’s consent to participate in the study. Eligible candidates were fully informed of what was expected of them, that their participation was entirely voluntary, and that they were free to withdraw from the study at any time without penalty.

### Data collection

Seven participants opted for an interview. The interviews, each lasting about 45 min, explored each participant’s gambling-related experiences during their childhood and adolescence. Participants were asked about their exposure to gambling, attitudes to and participation in gambling while growing up, factors that facilitated or hindered any gambling, motivations for not currently gambling, the impacts of gambling on their lives, their family and social environments, their experiences with simulated gambling, and protective factors. Supplement A contains the full list of questions. Participants were compensated with an AU$60 GiftPay voucher.

Seven additional participants participated in an online community. The online community was convened over seven days, using the Visions Live platform which resembles a social media platform. Participants were asked to participate for about one hour each day in activities and discussions designed to capture their gambling-related experiences while growing up. Nine topics were covered: (1) gambling behaviours and attitudes; (2) parental and family gambling attitudes and behaviours; (3) peer influence; (4) gaming and simulated gambling; (5) their ‘gambling journey’, including key milestones and influences over time; (6) gambling advertising; (7) gambling harms; (8) protective strategies; and (9) future gambling intentions. Supplement B contains the full list of questions. All participants used anonymous avatars. Tiered compensation was based on the number of days they participated, with a maximum of AU$140 in GiftPay vouchers available.

Individual interviews enabled an in-depth oral and narrative account of developmental influences on each participant’s choice to not gamble, while the online communities enabled participants to consider their answers over a more extended time period, to share information on sensitive topics in an anonymous way, and to discuss the topics with the other participants. While the format of questions was adapted to suit the conversational vs. written format of these activities, all were designed to address the same research aims so the two datasets were combined for analysis.

### Analysis

An adaptive grounded theory method was used which combines inductive and deductive analysis [25]. We used inductive methods to initially openly code and analyse emergent findings from the data, which were also informed by the literature review on sources of influence on young people’s gambling (parents, peers, marketing, etc.). After data familiarisation, we used the constant comparative method to code phrases, sentences and paragraphs in the data to identify relevant features, refine the codes as the analysis progressed, and group and collapse similar codes into broader themes. For example, codes related to ‘parents not gambling,’ ‘parents talking about gambling risks and harm,’ and ‘parental restrictions’ were grouped into a broader theme of ‘parental modelling, rules and guidance shape gambling attitudes and behaviours.’ Deductive consolidation of themes into multiple levels of influence was informed by a public health, socio-ecological systems approach [12, 13] to understand the complex multifaceted nature of factors that contribute to adolescent gambling beliefs, behaviours and attitudes. This process allowed us to identify meaningful patterns in the data. While there were some differences in wording and phrasing of codes between the researchers at the preliminary, inductive stages of data analysis, there were no conflicts when consolidating and coding themes in later stages of the analysis.

Trustworthiness of the research was enhanced by collecting data from participants with lived experience, using open-ended questions, and allowing participants to have control over the experiences they shared. Multiple researchers reviewed each analysis draft to ensure confirmability. Participants’ quotes increase authenticity. These are tagged by gender (male, female), age group in years (12–14, 15–17), and data collection method (IDI = interviews, OLC = online community).

### Findings

**Fig. 1:**
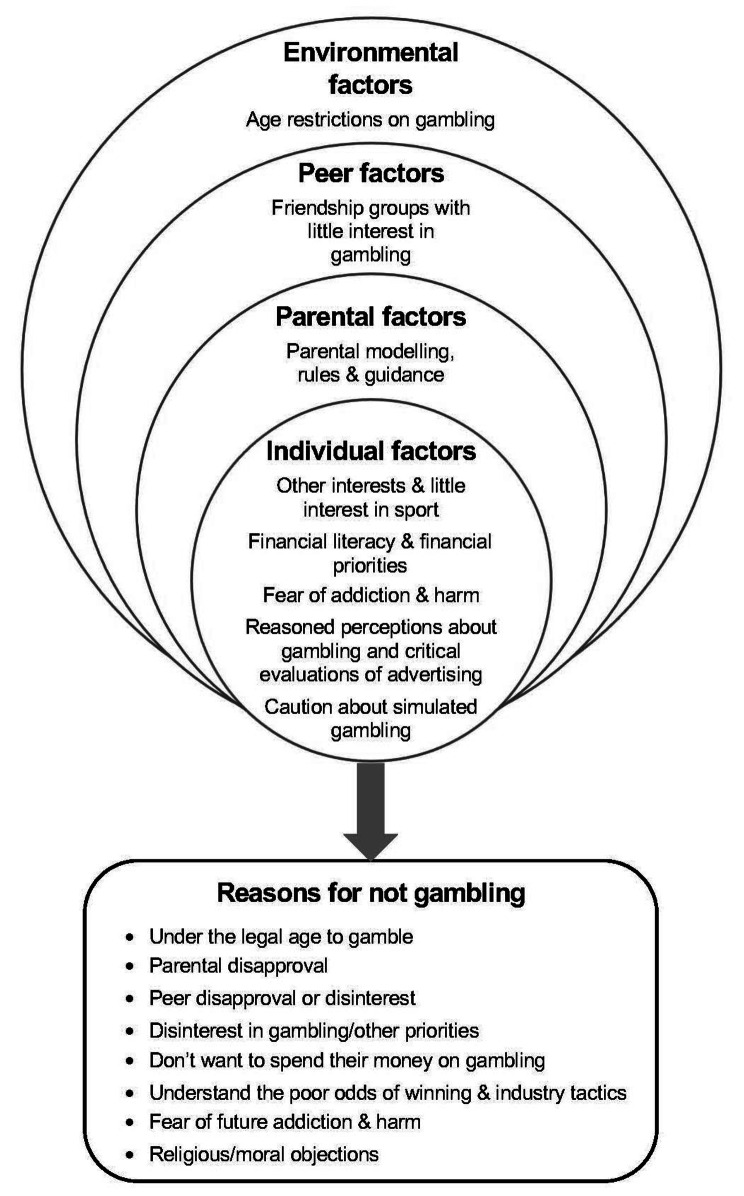
Social determinants of reasons for not gambling amongst adolescents

Eight themes emerged from the analysis that were grouped into four socio-ecological levels (Fig. 1). Environmental influences that shaped reasons for not gambling included age restrictions on gambling. Peer influences comprised having friendship groups with little interest in gambling. Parental influences entailed parental modelling, rules and guidance. Individual factors included having other interests and having little interest in sport, financial literacy and financial priorities, fear of addiction and harmful consequences, reasoned perceptions about gambling and critical evaluations of advertising, and caution about simulated gambling. These influences underpinned several reasons for not gambling articulated by the participants (Fig. 1).

### Age restrictions are seen as an unequivocal barrier to gambling

In Australia, it is illegal for people under 18 years to gamble on commercial gambling products. Nearly all participants were quick to note that being under the legal gambling age was the most obvious deterrent to them gambling. They appeared to accept these age restrictions as an unequivocal barrier, based on an implicit trust that the rules exist for a reason: ‘I always… thought that it’s a grown-up thing’ (#1, male, 15–17, IDI). No participants indicated any interest in circumventing age requirements for gambling, even though this was said to be easy:

[Young people] probably could easily get a fake licence or ID, could probably influence an adult or an adult wants to let them into this… [and] some places don’t have the best security in the front entrances, so someone could probably sneak in if they looked a bit older. (#8, male, 12–14, OLC)

Participating only in age-appropriate activities was also an expectation set out by their parents. These young people appeared eager to meet their parents’ expectations and to not break any rules. Accepting that gambling when underage was forbidden was said to lower their interest in gambling.

I don’t gamble because I don’t find it interesting and it is illegal for someone my age, my parents would not want me to gamble. (#13, female, 15–17, OLC)

I’ve always been told to not go anywhere near it. I mean I’m also underage so not allowed to, but then it’s also like I’ve always been told that it’s bad and that you could lose a lot of money. (#4, female, 15–17, IDI.)

### Parental modelling, rules and guidance shape gambling attitudes and behaviours

In the current study, parental influence was said to be critically important in shaping the participants’ gambling attitudes and behaviours from early childhood onwards. Most participants reported that their parents did not gamble or did so only occasionally. This limited parental gambling was usually associated with having negative opinions of gambling which, in turn, were said to shape the young person’s attitudes and behaviours.

My parents always despised gambling as my uncle wasted all his money on it and went off the rails. So that early instilling of the bad rep of gambling has stuck with me. (#10, male, 15–17, OLC)

I think that my parents don’t gamble, and don’t have anything good to say about gambling, has influenced me a lot… Parents think it’s a waste of money as much more likely to lose money than win it… it makes me feel like it’s all fake and everyone who goes there comes back home with empty pockets. (#12, female, 12–14, OLC)

Because the participants tended to recognise how their parents’ opinions, advice and behaviour have influenced their own aversion to gambling, some were highly critical of parents who gambled in front of children.

It sucks that people think it’s ok to do this kind of stuff around kids, who are largely influenced by their parents, as they will view them as heroic figures, and will adopt these bad traits onto themselves. (#8, male, 12–14, OLC)

As well as protecting their child from socialisation into gambling through the family, educating them on the risks and harms of gambling was another protective parental influence that participants recalled. They typically recounted that early childhood messages from their parents focused mostly on conveying a general disapproval of gambling, and then progressed to more detailed conversations about gambling risks and harms as the participants became older. They particularly remembered the cautionary tales that their parents related, usually during the participants’ early adolescence when their exposure to gambling was increasing. These conversations were often reactive, in response to an external cue such as a gambling advertisement. Participants recalled being especially responsive to stories based on real experiences.

My mum is a police officer, so I’ve heard… stories about the dark sides of gambling… and getting addicted to it… [Gambling] hasn’t really interested me that much because I know what can go wrong. (#1, male, 15–17, IDI)

Some participants reported that witnessing harm from gambling made an impression by raising their awareness of the likelihood of gambling losses and the risk of addiction.

I know now that… you’re more likely to lose lots of money than win lots of money… when I saw my Pop losing heaps of money, I’m like, ‘Oh, it’s not all win, win, win.’ (#2, male, 12–14, IDI).

Going to Las Vegas, seeing people betting and all the machines… It made me realise how addicted people are. (#12, female, 12–14, OLC)

On a school excursion, we had a guest speaker who had experienced gambling… he had taken money out of his workplace… then gambled the money… then he was trying to get it back through gambling… his experience of how that really forced him to experience a lot of hardship with his family and trying to find support with that. So, I’d seen, through those kind of things, the ways that it can negatively impact on people and the way that you can lose control. (#5, female, 15–17, IDI)

Most participants reported parental monitoring and control over their gambling, online gaming and simulated gambling. One participant described how his parents had a ‘no gambling’ rule, and another reported that his mother monitored and limited his spending on in-game items when playing video games. Some parents were also aware of simulated gambling elements in online games and were cautious about their child’s engagement.

It looked like a pokies machine. That’s why my mum was concerned with me playing it because you pulled down the lever and the thing spun, and then if you collected three of those things then you got a reward. (#4, female, 15–17, IDI)

### Protective influences from friendship groups with little interest in gambling

As young people enter and advance through their teenage years, peer influences on gambling tend to become more significant. However, while the participants recognised that peer influences could encourage gambling, most reported that their friends did not gamble or that gambling was not part of the interests, activities or conversations in their friendship groups: ‘Me and my friends never really bring up the topic “gambling” and I have never seen them talk about it to anyone else’ (#11, female, 12–14, OLC).

One participant explained that the moral values associated with her cultural background were her main deterrent. Having friends with a similar background also limited her interest in gambling because this friendship group shared other hobbies.

My friends come from backgrounds where gambling is highly discouraged and they have carried that out through our friendship, we don’t talk about gambling often and so I tend not to associate with it, this has also discouraged me from gambling. We have other interests and activities to do that don’t involve gambling. (#13, female, 15–17, OLC)

Peers were also said to influence the participants’ attitudes to gambling through vicarious experiences of gambling losses. For example, this participant reported that seeing or hearing about friends losing increased his awareness of the negative consequences that gambling could have: ‘I saw my friends… if they lost then they’d be all like upset… so I started to see like the downsides of it as well’ (#1, male, 15–17, IDI). Some older participants noticed increased peer involvement in gambling in their later teens, alongside more opportunities to gamble. However, the attendant risks appeared to be offset by other environmental, parental and peer protective factors.

### Having other interests, and little interest in sport

Many participants discussed how having other hobbies and activities left them with little time or interest in gambling. These activities included dancing, painting, drawing, music and skateboarding, which they might do alone or with friends: ‘My activities outside of school keep me occupied and less likely to take an interest in gambling’ (#12, female, 12–14, OLC). Alternatively, some participants commented that gambling could distract young people from more productive interests and pursuits. Participants recognised that having gambling-related interests might override an adolescent’s interest in other activities, including schoolwork: ‘People start gambling from a young age and set this as their future job [instead] of… focusing on school and their studies and setting a good career’ (#11, female, 12–14, OLC).

Further, an interest in following professional sport was said to expose young people to gambling influences and act as a ‘gateway’ to an interest in gambling. Some participants commented that their own lack of interest in sport helped to protect them from frequent exposure to betting influences and activities. They did not see the point in betting on sporting competitions that they had no interest in. Other participants did report an interest in sport but resisted its gambling influences, possibly due to other protective factors such as parental influences.

### Financial literacy and financial priorities

Numerous participants referred to gambling as ‘a waste of money’, a view most said had been conveyed by their parents. These adolescents did not see the point of engaging in chance activities where they risked losing their money: ‘Why waste your money on something that won’t necessarily work?’ (#14, female, 15–17, OLC). Several explained they understood there was a greater chance of losing than winning.

If I were to work hard every day, I would not want to waste it on a low chance of winning more and a high chance of losing most of my money… The closest thing I have done to gambling is just carnival stuff. (#8, male, 12–14, OLC)

These participants typically reported they had better things to spend their money on, both now and in the future. Older participants, in particular, appeared to have a well-developed sense of financial literacy, financial responsibility and future orientation. They believed that their appreciation of the value of money had been instilled by their parents. The following participant’s views on money demonstrate her high level of financial responsibility and her financial priorities that discouraged her from gambling.

I’m very like cautious about where my money goes… I don’t want to lose a lot of money because I like to save all of my money… I very much like to keep my money, because I love to travel and at the end of school, I want to travel around the world a bit. And then I also need to save up for uni and everything, because I don’t want to have a lot of debts… I very much like to know where my money is… Because money is very valuable, especially now when houses cost like tonnes of money, and you need to save up to buy a lot of things, and like inflation is making things more expensive. (#4, female, 15–17, IDI)

### Fear of addiction and harmful consequences

Participants reported that fear of addiction and the negative consequences of gambling were powerful deterrents. They recognised a wide range of potential harms, including to finances, relationships, mental health, anti-social behaviour and vocational performance.

Gambling at this age can also lead to higher rates of depression and anxiety, loss of friendship with non-gambling peers, and can also take you away from your family… taking money from your parents, changes in sleep patterns, low energy levels, changes in mood, and can be involved in risk-taking behaviour like fights, vandalism or shoplifting. (#11, female, 12–14, OLC)

Getting addicted to it and losing a lot of money… using possessions and stuff even, betting those when you have nothing left even. Like, it’s just like a hole (#1, male, 15–17, IDI).

There was widespread recognition in this cohort that, while gambling harm could be immediate, it could also have long-lasting impacts. Participants tended to view the harm from gambling as extreme and potentially life-changing: ‘it can ruin lives and families, it puts people in debt and ruins whatever they have built their life up to’ (#8, male, 12–14, OLC).

### Reasoned perceptions about gambling and critical evaluations of advertising

Amongst the participants, rational beliefs about gambling were evident, particularly in their understanding of the relative chances of winning and losing. Even though some acknowledged the appeal of gambling, they resisted its excitement and financial opportunity because they were aware of the likelihood of losing.

I can see how gambling might be fun due to the adrenaline it can produce or the money which someone could gain, but in my opinion the risk is not worth it. (#9, male, 15–17, OLC)

More commonly, participants said that they were just not interested in gambling, which they often attributed to their rational mindset and ability to think critically, as well as parental advice on how gambling works.

I’m kind of a person who’s very interested in things… ‘So how does it work? What are the odds– how like the statistically point whatever percent of people win something?’ And dad will bring up those things and you go, ‘Why do people even play that? It just seems silly’… When you’re saying there’s an opportunity to get millions of dollars, you’d be like, ‘Of course I want that.’ But… ‘what are the odds of that?’ It’s pretty slim. (#5, female, 15–17, IDI)

Some participants noted an increased awareness of gambling risks and harms as they got older, due to their increased “mental capability” (#5, female, 15–17, IDI). Several participants reported that they applied their critical thinking skills when considering the design of gambling products and their marketing. They felt in control of their choices and able to see through promotional messages about gambling. Two participants mentioned an interest in the psychology of advertising, which they felt helped them resist the appeal of gambling.

As a design student, and looking at the way designers and marketers will try and advertise and appeal to people, I think it’s allowed me to pick up on those things and understand why they’re doing some of the things they’re doing to try and engage an audience in a certain way. (#5, female, 15–17, IDI)

These participants also recognised that the design of gambling environments, including their sounds, lights and colours, is an industry tactic to encourage people to gamble. Some participants recalled being attracted to and intrigued by the design features of gaming rooms they saw as children when they dined at a venue with their family.

I could hear the noises, and I could hear, like, the sounds of the money… then when people opened the doors, I saw the colourful lights and I was, like, ‘Oh, I want to go in there,’ because, you know, I was a kid– it’s colourful. (#4, female, 15–17, IDI)

### Caution about simulated gambling

Like most young people, many participants regularly played video games, including games with simulated gambling elements such as loot boxes and wheel spinning. However, they tended to view spending real money in games, including on simulated gambling features, with a great deal of caution and had very low expectations of a worthwhile return. Some also recognised the potential for addiction to gaming and that simulated gambling could encourage young people to engage in monetary gambling.

Spending real money for skins and things is practically gambling… By spending money on skins and things worth no real-life value, the same person might be interested in spending money gambling with the chance to get real life money… A great example is the FIFA video game franchise, whereby you can either purchase ‘packs’ with an in-game currency or real money. Many of my friends decided to use in-game currency until they ran out but by then they were hooked and resorted to using their real money. (#9, male, 15–17, OLC)

The participants reported that their engagement in simulated gambling had not aroused temptations to engage in monetary gambling. However, they believed that other young people might not be so resistant. They saw the potential for simulated gambling to be a ‘gateway’ to real-world gambling, and that its heavy marketing and targeting of young people were harmful. Many were highly critical of the proliferation and extensive advertising of simulated gambling games, including through sponsored online influencers who typically show young people winning on these games in order to encourage real-money expenditure and persistent play.

They show in ads all the time, people just winning constantly but never really show how much money people use and how they get nothing in return and somehow people fall for the trick thinking that they will get loaded with money. I think the game/apps are worse because it shows that they’re winning a lot, which makes people play it more and that’s when the addiction begins. (#12, female, 12–14, OLC)

### Grounded theory model

Figure 1 presents the grounded theory model derived from the study’s findings. Key findings are discussed below.

## Discussion

This study has provided insights into the lived experiences of adolescents who refrain from gambling and how numerous social determinants when growing up interact to shape their reasons and choices to not gamble. As Fig. 1 indicates, the participants’ accounts highlight several reasons for not gambling. This study, and previous research, identify not being interested in gambling, being below the legal gambling age, discouragement from parent and peers, concern about gambling addiction and harm, not wanting to risk money on a low chance of winning, and moral objections, as reasons that some young people do not gamble [7, 18]. Unlike earlier research, however, no participants cited lack of access or opportunity as a reason for refraining from gambling. This may reflect the widespread availability of gambling in Australia, including through online and mobile devices and thousands of land-based venues, and opportunities to engage in private gambling.

Figure 1 also identifies several social determinants that provide deeper insights into factors that underpin the participants’ reasons for not gambling. In line with a socio-ecological perspective on health behaviour [12, 13], these social determinants include multiple layers of influence.

Parental factors appear to be the main formative influence on the participants’ gambling. Research has consistently found that parents play a crucial role in transferring gambling attitudes, knowledge and skills to their children, in educating them on the risks and harms of gambling, and in restricting their gambling and online activities [2, 8, 26]. Qualitative research has drawn on social learning theory to explain how parents can transfer knowledge and skills to their children, so that they learn how to gamble and assign positive meanings to the activity [27–28]. The current study shows how parents can have a converse effect through role modelling and other protective influences that deter their children from gambling. Parents were said to convey negative attitudes towards gambling, discourage gambling by their children, engage in no or limited gambling themselves, and advise their adolescents on the negative consequences of gambling. By limiting their own gambling, these parents helped to protect their children from being exposed to and involved in gambling, and from learning to gamble during childhood [27, 28]). Moreover, while harmful parental gambling increases the risk of gambling problems in children [8, 29], being exposed to harmful consequences in others, outside the nuclear family, may instead have an educative effect.

Social learning also occurs through peers, particularly in early and later adolescence, when friendship groups can introduce young people to gambling activities, encourage them to gamble, and provide the social rewards of in-group status and peer bonding [30, 31]. Peers can influence an adolescent’s gambling behaviour, depending on how normalised, encouraged or discouraged gambling is in their social group [30]. The current research found that when gambling is not an accepted or shared activity in friendship groups, peers can be a discouraging influence on gambling through their disapproval and avoidance of gambling and by sharing other non-gambling interests.

Environmental factors also shape youth gambling behaviour. Age restrictions on gambling are an important deterrent, as found in this and previous research [7]. While these age restrictions apply to all underage adolescents, the non-gamblers in this study accepted them as an unequivocal barrier, even though other adolescents might choose to circumvent them. This suggests that it is not just the presence of these restrictions, but instead how young people respond to them, that impacts on their subsequent gambling involvement. These responses may reflect more generalised attitudes to compliance with rules and parental restrictions. Nonetheless, better enforcement of age and identity requirements may further assist in preventing gambling by minors, given that underage access to some commercial gambling products is reportedly easy [9].

Previous studies have also examined other environmental influences on youth gambling, although mainly in relation to those that encourage gambling. A key focus has been on the role of advertising in fostering youth gambling [8, 32–34] and how simulated gambling can normalise and be a training ground for monetary gambling [35, 36]. Like most young people in Australia, the adolescent non-gamblers in this study reported widespread exposure to gambling advertising and simulated gambling [8, 20]. However, many explained they were sceptical about gambling marketing claims and cautious about simulated gambling, particularly spending real money on this activity. This reasoned and critical thinking about industry tactics and the odds of winning were said to temper their responses to these marketing influences. Other Australian research has found that children are exposed to and can recall the sights and sounds of gambling in venues, even when gambling products are in restricted areas [37, 38].

Social connectedness, fostered by extracurricular activities, positive parent-child relationships and pro-social behaviour, is said to lower the likelihood of youth gambling [2]. Many participants also had little interest in professional sport, so they may be somewhat protected from the associated advertising and other gambling influences that occur when people watch sports broadcasts and share an interest in sport with family and friends [39, 40]. Consistent with previous research [41], having gambling-related interests might override an adolescent’s interest in other activities, including schoolwork. However, it is unclear whether the social connectedness and diversion of having other hobbies and interests is a cause, consequence or co-occurring feature of gambling involvement. Research into adolescents who watch sports but do not gamble is required to better understand factors that help them resist gambling influences in this context.

Several individual factors were implicated in the reasons these young people refrained from gambling which, in turn, may have been shaped by factors such as their personality, parental discipline and friendship groups. Aligned with their tendency for reasoned and critical thinking, the participants saw gambling as a waste of money because of the low chances of winning. Instead, they prioritised spending their money on other interests or tangible goods, and older participants tended to have savings goals for future acquisitions and activities. Research has consistently found a significant relationship between erroneous gambling cognitions and gambling problems in youth and, conversely, the protective influence of rational gambling beliefs [42–45]. Financial literacy, that is, being able to make effective decisions about expenditure, saving and budgeting, has an inverse relationship with gambling frequency [46, 47], and may therefore deter young people from gambling. The participants’ awareness that people are most likely to lose at gambling was often instilled by parents, who also conveyed cautionary tales and guidance that gambling could lead to addiction and harmful consequences. These young people appeared to take these messages seriously and were fearful that gambling would lead to life-changing harms. Participants recognised a wide range of potential harms, including to finances, relationships, mental health, anti-social behaviour and vocational performance, as also identified in models of gambling harm [15, 48, 49]. Overall, the participants indicated little interest in gambling and instead reported having a wide variety of other interests that they pursued alone or with family and friends. This aligns with previous findings that extra-curricular activities and social connectedness are protective influences for youth gambling [2].

Several implications arise from the study’s findings. Protective factors implicated in the participants’ reasons and choices to not gamble emanated from multiple layers of influence. This implies that multi-layer interventions, in line with a public health response, are likely to be optimal in deterring underage gambling– including to young people who already gamble and are likely to experience gambling harm. While not all risk and protective factors for gambling and gambling harm are modifiable, those suggested here are practical strategies aimed at preventing and reducing harm amongst young people. At the environmental level, better age-gating for both monetary and simulated gambling, along with less exposure of children to promotional gambling messages, can help protect to young people who might otherwise struggle to resist these influences. Since young people are less able to critically assess gambling marketing, regulation to prevent the advertising of gambling to children and adolescents is a vital strategy [50]. Interventions that support parental role modelling and guidance for their children can include raising awareness about how parents influence their children’s gambling, and the provision of advice and resources they can use to deter them [27, 51].

Youth education is also needed using evidence-based programs. The current study indicates that potentially useful elements include cautionary tales based on the lived experience of people harmed by gambling, as well as education to increase young people’s financial literacy, ability to recognise marketing tactics, awareness of the risks and harms of gambling, and how they might resist peer and other normalising gambling influences. Youth gambling education programs should also be informed by previous research evidence. For example, a systematic review of behavioural change techniques directed at youth gambling indicate that the most successful programs include information of the harm from gambling to relationships, finances, and mental health [52]. Donati et al. [44] found that a brief, online, school-based psychoeducational intervention, that comprised a gambling-specific skills training program, increased awareness about gambling, undermined gambling-related cognitive distortions, and reduced gambling frequency and gambling problems.

Naturally, this study has limitations. It focused on gathering in-depth information to provide detailed insights into the lived experiences of a small sample of participants, so the findings may not be generalisable to all adolescent non-gamblers. Data saturation may not have been achieved, and future research could obtain larger samples. The findings may also be influenced by social desirability bias and recall bias, although the memories people have and how they interpret them are likely to influence their subsequent attitudes and behaviours. Given that participants were compensated for their time, the sample may also be skewed towards adolescents who had a greater need for money. A self-selection bias due to the need for parental consent may be present, as consenting parents may have attitudes to gambling and parenting approaches that differ from the broader population. Future research could examine whether this bias exists and how differences in parent-child relationships and attachment styles affect the learning about gambling that occurs in childhood and adolescence. It is also possible that some parents may have monitored their child’s responses to the OLC activities and impacted their responses, or that OLC participants sourced other information to inform their responses.

Future research could collect more detailed demographic data to better understand how adolescents’ decision to not gamble intersect with factors such as socioeconomic status, family circumstances, health, ethnicity, religious beliefs, and school grades, which have been implicated in pathways into gambling and gambling harm [29, 53–58].

Grounded theory methodology is necessarily subjective in nature, with the findings shaped by how participants interpret and share their experiences and how the researchers interpret the data. While generalisability is therefore limited, the current study helps to advance understanding beyond simple self-reported reasons for not gambling to identify multi-layered social determinants and processes that can underpin these reasons amongst young people. The resulting grounded theory can inform protective strategies and further research. This study found that the most potent social determinants of non-gambling were from the individual, parental and peer levels. Research that explores how social and commercial determinants at the community, systems, industry and societal levels impact on young people’s gambling choices would also be valuable.

## Conclusion

This study has provided a detailed exploration of adolescent non-gamblers and how their reasons and choices to not gamble are shaped by social determinants as they grow up. It concludes that multiple factors and layers of influence interact to deter young people from underage gambling. While the environmental factor of age restrictions on gambling is an important deterrent, parental influences through appropriate role modelling, rules and guidance, as well as peer influences from friendship groups with little interest in gambling, appear to be stronger influences. These influences shape and interact with individual factors to act as deterrents to gambling. Individual factors include having other interests, little interest in sport, financial priorities, fear of addiction and harm from both gambling and simulated gambling, reasoned perceptions about gambling, and the ability to critically evaluate gambling advertising. Research into adolescent gambling lacks a focus on interventions to reduce gambling harm [2]. The present findings, therefore, contribute knowledge to inform preventive strategies such as youth education programs and parental resources and support, that can help to deter underage gambling through reducing modifiable risk factors and enhancing modifiable protective factors. Importantly however, strategies are also needed to reduce environmental risk factors for gambling harm, such as widespread child exposure to gambling advertising and the normalising influences from simulated gambling. Since early uptake of gambling increases the risk of harmful gambling and subsequent mental disorders in later life [4], multi-layered public health interventions are important to discourage gambling in adolescents. This exploratory study has provided some preliminary insights into the social determinants that shape some adolescents’ reasons for not gambling, but further research is needed to optimise evidence-based interventions.

### Electronic supplementary material

Below is the link to the electronic supplementary material.

Supplementary Material 1

Supplementary Material 2

## Data Availability

The data that support the findings of this study are available from the New South Wales Office of Responsible Gambling but restrictions apply to the availability of these data, which were used under license for the current study, and so are not publicly available. Data are however available from the authors upon reasonable request and with permission of the New South Wales Office of Responsible Gambling. In the first instance, please contact the corresponding author, Nerilee Hing: n.hing@cqu.edu.au.
