# Supplementary material for: ‘Getting addicted to it and losing a lot of money… it’s just like a hole.’ A grounded theory model of how social determinants shape adolescents’ choices to not gamble
Source: BMC Public Health. 2024 May 9;24:1270. doi: 10.1186/s12889-024-18286-3 (PMC11084133; doi:10.1186/s12889-024-18286-3)
Supplement: Supplementary file 1 — Supplementary Material 1 [file 12889_2024_18286_MOESM1_ESM.docx]

**Supplement A: Interview Questions**

**FIRST EXPERIENCES AND MEMORIES OF GAMBLING**

- Thinking back to when you were young, what are your first memories of gambling? When did you first become aware that gambling exists? What was the first type of gambling you became aware of? How do you think you first become aware of gambling (e.g., parents, friends, advertising, video games)?

**CHILDHOOD (up to 10 years old)**

The next questions are about when you were aged 10 years or younger. You would have been in primary school then, and probably turned 10 in Grade 4 or 5.

- **Awareness of gambling**. Thinking back to your early years when you were 10 or younger and in primary school, what type of gambling were you most aware of? What other types of gambling were you aware of?
- **Parents’ behaviours and attitudes to gambling**. When you were 10 or younger, did your parents gamble? Did they ever talk about gambling or gamble in front of you? Or involve you in gambling? Please tell me what you remember.
- **Others’ behaviours and attitudes to gambling**. When you were 10 or younger, did any other people around you gamble, e.g., friends, other family members? Did they ever talk about gambling or gamble in front of you? Or involve you in gambling? Please tell me what you remember.
- **Exposure to gambling advertising**. When you were 10 or younger, do you recall seeing any gambling advertising? Please tell me what you remember. Did it make you interested in gambling? Why/why not?
- **Exposure to games with gambling components**. Some video games have gambling components, that look and play like normal gambling games, but you cannot win real money. These can include 1) games with ‘mini’ gambling activities in them (e.g., wheel spinning, slots), 2) social casino games in apps and social media (like Zynga games on Facebook and poker, slots and bingo apps from an app store), 3) demo or practice games on real gambling websites, and 4) loot boxes. When you were 10 or younger, did you play any games with these types of gambling components? Please tell me what you remember about this, e.g., types of gambling components in games, how often you played them, if you spent money on them, what you thought of them. Did they make you interested in gambling for money? Why/why not?
- **Own attitudes to gambling**. When you were 10 or younger, what did you think of gambling? Did you think it was a good thing, perhaps exciting, or perhaps boring or a bad thing? Why did you feel this way?
- **Own participation in gambling**. When you were 10 or younger, did you ever gamble yourself? Please tell me what you remember about this, e.g., what did you gamble on, how often, did you spend much money, did you do this alone or with other people, who? What were your main reasons for gambling/not gambling?
- **Changes in own gambling**. When you were 10 or younger, did your gambling change at all e.g., started gambling, increased, decreased, stayed the same. Please tell me more about this. What do you think were the main reasons for this?

**EARLY ADOLESCENCE (11-14 years old)**

Now I’d like to ask you about when you were aged between 11 and 14 years. You would probably have been in the last year of primary school and early years of high school, so around Grades 6 to 9.

- **Awareness of gambling**. Thinking back to your early teenage years when you were 11 to 14 years old, what type of gambling were you most aware of? What other types of gambling were you aware of?
- **Parents’ behaviours and attitudes to gambling**. When you were 11 to 14 years old, did your parents gamble? Did they ever talk about gambling or gamble in front of you? Or involve you in gambling? Please tell me what you remember.
- **Others’ behaviours and attitudes to gambling**. When you were 11 to 14 years old, did any other people around you gamble, e.g., friends, other family members? Did they ever talk about gambling or gamble in front of you? Or involve you in gambling? Please tell me what you remember.
- **Exposure to gambling advertising**. When you were 11 to 14 years old, do you recall seeing any gambling advertising? Please tell me what you remember. Did it make you interested in gambling? Why/why not?
- **Exposure to games with gambling components**. Earlier, we talked about games with gambling components. These are games that look and play like normal gambling games, but you cannot win real money. When you were 11 to 14 years old, did you play any of these games? Please tell me what you remember about this, e.g., types of gambling components in games, how often you played them, if you spent money on them, what you thought of them. Did they make you interested in gambling for money? Why/why not?
- **Own attitudes to gambling**. When you were 11 to 14 years old, what did you think of gambling? Did you think it was a good thing, perhaps exciting, or perhaps boring or a bad thing? Why did you feel this way?
- **Own participation in gambling**. When you were 11 to 14 years old, did you ever gamble yourself? Please tell me what you remember about this, e.g., what did you gamble on, how often, did you spend much money, did you do this alone or with other people, who? What were your main reasons for gambling/not gambling?
- **Opportunities to gamble**. When you were 11 to 14 years old, would you have been able to gamble if you wanted to? Did you have access to any gambling, any money to spend on gambling, or opportunities to gamble with others?
- **Barriers to gambling**. When you were 11 to 14 years old, did some things discourage you from gambling, e.g., parental rules, parental disapproval, awareness of gambling harm, lack of interest?
- **Changes in own gambling**. When you were 11 to 14 years old, did your gambling change at all e.g., started gambling, increased, decreased, stayed the same. Please tell me more about this. What do you think were the main reasons for this?

**LATE ADOLESCENCE (15-17 years old)**

Now I’d like to ask you about when you were aged between 15 and 17 years. You would probably have been in senior high school, so around Grades 10 to 12.

- **Awareness of gambling**. Thinking back to your later teenage years when you were 15 to 17 years old, what type of gambling were you most aware of? What other types of gambling were you aware of?
- **Parents’ behaviours and attitudes to gambling**. When you were 15 to 17 years old, did your parents gamble? Did they ever talk about gambling or gamble in front of you? Or involve you in gambling? Please tell me what you remember.
- **Others’ behaviours and attitudes to gambling**. When you were 15 to 17 years old, did any other people around you gamble, e.g., friends, other family members? Did they ever talk about gambling or gamble in front of you? Or involve you in gambling? Please tell me what you remember.
- **Exposure to gambling advertising**. When you were 15 to 17 years old, do you recall seeing any gambling advertising? Please tell me what you remember. Did it make you interested in gambling? Why/why not?
- **Exposure to games with gambling components**. Earlier, we talked about games with gambling components. These are games that look and play like normal gambling games, but you cannot win real money. When you were 15 to 17 years old, did you play any of these games? Please tell me what you remember about this, e.g., types of gambling components in games, how often you played them, if you spent money on them, what you thought of them. Did they make you interested in gambling for money? Why/why not?
- **Own attitudes to gambling**. When you were 15 to 17 years old, what did you think of gambling? Did you think it was a good thing, perhaps exciting, or perhaps boring or a bad thing? Why did you feel this way?
- **Own participation in gambling**. When you were 15 to 17 years old, did you ever gamble yourself? Please tell me what you remember about this, e.g., what did you gamble on, how often, did you spend much money, did you do this alone or with other people, who? What were your main reasons for gambling/not gambling?
- **Opportunities to gamble**. When you were 15 to 17 years old, would you have been able to gamble if you wanted to? Did you have access to any gambling, any money to spend on gambling, or opportunities to gamble with others?
- **Barriers to gambling**. When you were 15 to 17 years old, did some things discourage you from gambling, e.g., parental rules, parental disapproval, awareness of gambling harm, lack of interest?
- **Changes in own gambling**. When you were 15 to 17 years old, did your gambling change at all e.g., started gambling, increased, decreased, stayed the same. Please tell me more about this. What do you think were the main reasons for this?

**FINAL QUESTIONS after all relevant time periods have been asked about:**

- **Is there anything else** that may have influenced your attitudes and behaviours towards gambling when you were growing up that you’d like to tell me about?
- **Harms from own gambling**. When you were growing up, what were the bad things, if any about your gambling that may have caused harm to yourself or others? What kinds of harm, e.g., to relationships, school, money, your health and wellbeing? Please tell me more about this.
- **Protective strategies and environments**. Some young people experience problems and harm from their gambling. What do you think could be done to better protect young people from gambling problems and harm? E.g., is there anything that could be done by parents, friends, schools, gambling operators, governments, help services, advertising?
