# Supplementary material for: ‘Getting addicted to it and losing a lot of money… it’s just like a hole.’ A grounded theory model of how social determinants shape adolescents’ choices to not gamble
Source: BMC Public Health. 2024 May 9;24:1270. doi: 10.1186/s12889-024-18286-3 (PMC11084133; doi:10.1186/s12889-024-18286-3)
Supplement: Supplementary file 2 — Supplementary Material 2 [file 12889_2024_18286_MOESM2_ESM.docx]

**Supplement B: Online Community Questions**

**Day 1**

- **Gambling in your eyes**

**What is 'gambling' to you**? 
Tell us what kind of activities you think count as gambling, and how you feel about gambling overall. 
*Answer questions like this by typing your response in the text box below and clicking 'submit'.  After you submit your answer, it will be visible to the rest of the Community and you can read other answers too. Some of the questions we'll ask this week will be a bit more private - we'll let you know when there's a task where your response WILL NOT be able to be viewed by other people.*

- **The more you know…**

When we mention 'gambling', we mean: Spending real money gambling online or in land-based venues (like in a pub or club) for a chance to win real money.
**Select all the types of gambling you've heard of before**: *Poker machines;* *Horse or greyhound races; Casino games, such as poker, blackjack or roulette; sporting events betting (including betting on football, soccer matches etc, but also on other events like reality TV outcomes); Esports events betting (competition using video games like League of Legends, Overwatch, Super Smash Bros, StarCraft); Fantasy sports games (imaginary or virtual teams like fantasy football); Scratchies, lottery or lotto tickets; Keno (you see this in pubs/clubs, a sheet of paper where you pick numbers and they are drawn, often over TV, and you see how many you got); Bingo; Private games, such as card or dice games with friends or family; Betting using skins or in-game items (such as clothes, weapons, appearance enhancements)*

- **List them out**

What, if any, types of gambling have you ever done where you’ve…

1.       Spent **real money** gambling online or in land-based venues (like a pub or club)

2.       For a chance to win **real money**

- **How often do you…**

**Overall, how often do you gamble now**?

*Select one: Often, Sometimes, Hardly ever, Never*

- **Why do you gamble**?

*Select all that apply:* *It is fun/exciting; It feels grown-up; It makes watching sport/playing games more exciting; To win money; It is a habit; To spend time with family; To fit in with friends; To spend time with friends; To pass the time when you’re bored; It is interesting/teaches you skills e.g. understanding odds; It takes your mind off problems and worries*

In the comments below, pick the one reason that **best** explains why you gamble and tell us a little more about it. Please include a story or example if you can!

- **Pick a side**

For the next few tasks, you will be shown two words or terms that are the opposite of each other, like fun vs boring. Choose the answer that is closest to how you feel about gambling: *Fun vs Boring; Glamorous vs Dull; Against the Rules vs Fun for All Ages; Based on Chance vs Based on Skill; High Chance of Winning vs Low Chance of Winning*

Now please **pick one word or term that you feel most strongly about**, and explain why to the rest of the Community (you can pick more than one if you like).

Compare your answers – see if you can find someone who picked the opposite word to you and why they feel that way! Respond to their post and let them know what you’re thinking.
(A reminder: the words to pick from are fun, boring, glamorous, dull, against the rules, fun for all ages, based on chance, based on skill, high chance of winning, low chance of winning)

- **Tell us why not**

You’ve mentioned that you do **very little, if any, gambling**.

Please take a minute to think about why that is and describe to us why you don’t gamble. 

- **What age were you when...**

We’re going to start you off with a timeline of some of your ‘firsts’ when it comes to gambling.

Below is a blank timeline with ages 4 to 17 and a group of numbered pins. Each pin represents a question (numbered below). **Drag each pin to the approximate age you were when you first experienced each of the events** below. Your best guess for your age is fine!  If there are any you haven't heard of or done before, just select the pin and click 'X' at the bottom of your whiteboard to delete.

1. First time you heard someone talk about gambling
2. First time you saw your parent/guardian or another family member gamble
3. First time you were involved in someone else’s gambling (such as picking a number or a team, scratching a scratchie)
4. First time you gambled yourself (i.e. you decided on the bet and the amount to spend)
5. First time you spent any of your own money on gambling
6. First time you gambled with friends or family for money or belongings (for example betting on a sports match, playing card games for money)
7. First time you remember gambling and winning
8. First time you remember gambling and losing
9. When you first became aware of gambling ads
10. First time you clicked on or followed a gambling advertisement to learn more or take up an offer

- **It made an impression!**

Now you're in the zone of thinking about when you were first exposed to gambling, can you tell us about **the most memorable first experiences you can recall about gambling**? 
We want to know what left a big impression on you as a child and shaped how you feel about gambling then and now. This might be one of the events we listed in the last question or something related to your family, friends etc.

- **What age were you when... (part 2)**

Still thinking about your 'firsts', **move the icon for each of the below types of gambling to the age you were when you first personally participated** in each.

In the comment below, tell us a bit more about your gambling experience, such as whether you did different types of gambling with parents or siblings, through an app or in venue etc. 
For any type of gambling you haven't tried, click on the icon and then click the 'X' button at the bottom of your whiteboard to delete it.

- **Gaming**

Some of the questions we ask you later in the week will be about **playing video or digital games**, so we’d like to get an idea of how involved you are in gaming.

**How often do you play online or video games** (including playing games on your phone, console etc)?

*Select one: Often; Sometimes; Hardly ever; Never*

**Day 2**

- **What do your parents think?**

Do your parents/guardians **approve or disapprove of gambling**?

*Select one: Strongly disapprove; Disapprove; Approve; Strongly approve*

- **What do your parents think?**

Do your parents/guardians approve of **your**gambling?

*Select one: No, not at all; They don’t like it, but allow it for special occasions; Yes, but in moderation; Yes, entirely; Your parents do not know that you gamble.*

- **What do your parents do?**

What **types of gambling do your parents/guardians do** (that you know about)?

*Select all that apply: Poker machines; Horse or greyhound races; Casino games, such as poker, blackjack or roulette; Sporting events; E sports events; Fantasy sports games; Scratchies, lottery or lotto tickets; Keno; Bingo; Private games, such as card or dice games with friends or family; Betting using skins or in-game items on any activity; Parents/guardians do not gamble*

In a comment below, tell us if your parents/guardians have always gambled, or if they have changed the way and amount they gamble over time*(think about this before COVID-19 if you can, as well as how their gambling changed during lockdown)*

- **How often do your parents…**

**How often do your parents/guardians gamble**?

Answer based on the parent/guardian who gambles the most often *Select one: Often; Sometimes; Hardly ever ; Never*

- **What you heard…**

These next activities ask you to tell us how parents/guardians or other adults:

1. **Talk about gambling in front of young people**
2. **Gamble in front of young people**
3. **Involve young people in gambling**

To answer these questions, we’d like you to talk about not just your own parents/guardians, but what you’ve seen with other families. Include any examples that stick out to you while you’ve been growing up.

**How do adults talk about gambling in front of young people?**

In your answer, tell us what kind of things about gambling you think is normal for adults to talk about in front of young people. Please include any examples you can think of with adults discussing gambling in front of young people (remember, gambling can be gambling can be sports or race betting, casino games, lotto, keno, bingo, pokies, etc.)

Respond to at least one other post from a Community member – you can add your own story to support their answer, or ask questions about their experiences.

- **What you saw…**

**How do adults gamble in front of young people?**

In your answer, tell us what kind of gambling you think is normal for young people to see adults doing. Please include any examples you can think of with adults gambling in front of young people (remember, gambling can be sports or race betting, casino games, lotto, keno, bingo, pokies etc. Remember, this is about your own family, other families and any stories you might have heard.

Respond to at least one other post from a Community member – you can add your own story to support their answer, or ask questions about their experiences.

- **and what you did**

**How do adults involve young people in gambling?**

In your answer, tell us what kind of gambling you think is normal for young people to be included in. Please include any examples you can think of with adults letting young people take part in gambling (remember, gambling can be sports or race betting, casino games, lotto, keno, bingo, pokies etc). Remember, this is about your own family, other families and any stories you might have heard.

Respond to at least one other post from a Community member – you can add your own story to support their answer, or ask questions about their experiences.

- **Family Matters**

Tell us about **anyone else in your family or household who gambled** when you've been growing up.

In your answer, please mention who they were (e.g. uncle, grandparent, cousin, brother), how they talked about gambling or gambled in front of you and whether you were involved in any of their gambling. Include examples if you can!

- **Under 18: Gambling methods**

Next, we’re going to discuss **how under 18s go about gambling**.

Using examples from your own life, stories you’ve heard from friends or people at school, post some of the ways teenagers gamble (and ways they might get around rules to gamble!)

In your answer, include**what kind of gambling** young people do, **where they get the money** to gamble, and **who they gamble with**.
*Reminder: the information you provide to us is confidential and anonymous.*

- **Cautionary tales**

**Have your parents/guardians** or other family members **ever talked with you about the risks and potential harms of gambling**? Answer below: *Yes, a lot; Yes, a little bit; No, never*

In the text box, tell us more about these conversations, if they happened. About how old were you when your parents/guardians first spoke to you about potential harms from gambling? What things did they talk about?

If not, why do you think you and your parents/guardians have never had that talk?

- **Family Influence**

How do the**views and behaviour of parents/guardians or other family affect young people’s views of gambling**? Post your answer and then have a look at answers from other people in the Community, and let them know if you agree with their thoughts.

- **Protective parents**

What do you think **parents/guardians or other family members can do to help make sure young people don’t gamble in a way that is harmful**?

Post one suggestion, then have a look at what other members of the Community are suggesting. Jump on to any threads you agree with and add what you can to the ideas until you think we, as a group, have a good list of what parents/guardians can/should do to support or protect young people.

**Day 3**

- **What are your friends up to?**

What types of gambling **do your friends do** (that you know about)? Remember! Gambling means spending **real money**.

*Select all the apply: Poker machines; Horse or greyhound races; Casino games, such as poker, blackjack or roulette; Sporting events; E sports events; Fantasy sports games; Scratchies, lottery or lotto tickets; Keno; Bingo; Private games, such as card or dice games with friends or family; Betting using skins or in-game items on any activity; None.*

- **May I introduce you to…**

Can you think of any examples of **a friend introducing a type of gambling to you** or to other young people you know? Tell us about it! Let us know if you’ve taught any of your friends anything about gambling.

- **We’re all in this together…?**

How does **a young person's friendship group affect their views of gambling and whether or not they gamble** themselves?

In your answer, think about examples from your own life and stories from friends or people at school. Respond to at least one other Community member’s answer you agree with and tell them why you agree.

- **Looking out for each other**

Is there anything you think**friends or peers should be doing to help make sure other young people don’t gamble in a way that is harmful**?

Post one suggestion, if you have any, then have a look at what other members of the Community are suggesting. Jump on to any threads you agree with and add what you can to the ideas until you think we, as a group, have a good list of what friends or peers can do to support or protect each other. 

- **Introduction**

Next we are going to talk about gambling-like games you play or have seen before.

**What are 'gambling-like games'?** You’ll find parts of video and online games that look like gambling. These games give you a chance of winning a prize (like in-game money, skins, or extra lives etc). Things like:

- Games which have**‘mini’ gambling activities within the game**, such as pokies/slot machines, wheel spinning or bingo. Example: the Diamond Casino & Resort in the video game Grand Theft Auto V.
- **Casino games that can be played on apps or social networking sites**. These look just like gambling activities, such as pokies/slot machines, roulette, blackjack. Example: Zynga games on Facebook or bingo, poker, pokies/slots or roulette that you can play on your phone, tablet or computer.
- **Practice or demo games on real gambling websites** **and apps**. Example: Mobile Casinos.
- **Loot boxes**where gamers can open an in-game digital container that contains a mystery item like clothing or weapons. Example: Minecraft, FIFA and many Roblox games.

But in these games or activities, you **cannot win real money**.

**How often do you play gambling-like games**

How **often do you come across gambling-like games**(now or in the last couple of years)?

*Please select one: Often; Sometimes; Hardly ever; Never*

- **What age were you when...**

The next question is just like the timeline of 'first times' you filled in on Monday - but now we want to ask you about 'gambling-like games' and the first times you saw or played games or activities that look like gambling. Remember, this includes games and features that LOOK like gambling, but you can't win real money. 
**Drag each pin to the approximate age you were when you first experienced each of the events** below. Your best guess for your age is fine!

If there are any you haven't heard of or done before, just select the pin and click 'X' at the bottom of your whiteboard to delete. First time you played a game that reminded you of gambling

1. First time you played a game that reminded you of gambling
2. First time you downloaded / purchased a game with gambling-like features
3. First time you won a good prize from gambling-like features (e.g. a high value loot box or rare skin)
4. First time you won a disappointing prize from gambling-like features (e.g. a bad loot box)
5. First time you saw an ad for a game that looked like it had gambling-like features

We've included an example for you below
In the comment below, you can**tell us a bit more about your experiences with gambling-like games and features**, like what types of gambling-like features (like loot boxes or spinning-the-wheel) you came across first, or memorable prizes or losses in these games

- **Pick a side**

For the next few tasks, you will be shown two words or terms that are the opposite of each other, like harmless vs harmful.

**Choose the answer that is closest to how you feel about gambling-like games, or gambling-like features within games you play**

*Select one: Glamorous vs Dull; Makes you feel lucky vs Makes you feel unlucky; Harmless vs Harmful; Based on Chance vs Based on Skill; The Odds of Winning are lower than in real money gambling vs The odds of winning are higher than in real money gambling; Makes the game more enjoyable vs Makes the game less enjoyable*

- **Which best describes your view of gambling-like games?**

Now please **pick one word or term that you feel most strongly about**, and explain why to the rest of the Community *(you can pick more than one if you like)*.
*(A reminder: the words to pick from are glamorous, dull, makes you feel lucky, makes you feel unlucky, harmless, harmful, based on chance, based on skill, high chance of winning, low chance of winning, makes the game more enjoyable and makes the game less enjoyable).*

- **Influence of apps**

Does playing games or apps that have **gambling-like features make young people more interested in trying gambling for real money**? Tell us whether you agree or disagree, and why. See if you can find someone in the Community who disagrees with you and (politely and respectfully) post a comment explaining why you feel differently. 

- **The money… it’s fake!**

What impact do you think **spending ‘fake’ money has on young people**? Do you think this could be**linked to gambling behaviour**?

- **Money money money**

Gambling-like games allow you to spend **real money**, for example to purchase loot boxes, buy in-game currency, etc. What impact do you think**spending real money in games has on young people**? Do you think this could be**linked to gambling behaviour**?
Please explain why you think this.

- **Crypto**

‘Crypto currency’ like bitcoin and Non-Fungible Tokens (NFTs) lets people buy digital  currencies and tokens with real money, hoping that their value goes up and they will make more money.
What impact do you think **the investment and trade of digital currencies (that don’t look like regular money) has on young people**? Does the way the value of these crypto currencies goes up and down r**emind you of gambling, or is it completely different**?
If you don’t understand what digital currencies are, that’s ok! Just let us know if you’ve seen or heard anyone talk about them, and if it reminded you of gambling or not.

- **The Rules of the Game**

Are there any**rules or restrictions for providing gambling-like features that game developers should have to follow**, in order to protect young people from developing harmful gambling behaviour?

Post your response with a rule or restriction you think they should follow (if any!). Have a look at what other members of the Community are suggesting, then add comments to other ideas until we, as a group, have a full list of rules we think are important to protect young people.

**Day 4**

- **Your Timeline**

Over the last few days, we’ve asked you to think a lot about gambling and gambling-like games and how they have appeared in your life. Now it’s time to bring all of this together!

Below is a blank timeline, the same as you filled out day one. We would like you to **drag each pin to the approximate age you were when you first experienced each of the events** below. Your best guess for you age is fine!*Please try to arrange your pins so they do not cross-over and are all clearly visible.*

Please do this **first for gambling-like games** (video or online games with gambling-like features where you can’t win real money)

  1. When you**started playing** any gambling-like games 
  2. When you were **playing the MOST gambling-like games**
  3. When you were**playing gambling-like games, but NOT very much**

Now please do the same for **gambling with real money**  4. When you were **gambling the MOST** (with real money)
  5. When you were **gambling, but NOT very much** (with real money)
  6. When you were really **enjoying**gambling
  7. When you were **NOT enjoying** gambling
  8-10 (use up to 3 pins). Any **big losses or wins** you can remember (for yourself, your family, or your friends)

You can use the comment box to tell us any important details, like why there was a time you were NOT enjoying gambling, or why you think you were playing the MOST gambling-like games at a certain age.

- **Just how fast the night changes…**

Take a minute to think about the timeline you’ve created.

Please spend the next few minutes explaining **any changes**there have been throughout your life**to how you gamble or play gambling-like games** (e.g. when your gambling went up or down or if you stopped gambling, if you were spending more at one time etc.) and **explain to us why**. What else was happening in your life?

If your timeline doesn’t show any changes, tell us about that! Why do you think the amount you gamble or play gambling-like games remains the same over time?

- **Number one, very important influencer**

Let’s focus just on gambling for**real money**. What, or who, do you think **had the biggest influence on your view of gambling and how you have gambled (or not)**?

Pick one of the following as your biggest influence and explain why, with examples if you can: P*arents/guardians; Other family members; Friends; Playing gambling-like games; Seeing or hearing about gambling elsewhere, like ads or movies; Other; Don't recall.*

- **Law & Order**

Are there any **rules or special laws that businesses, websites or apps that provide gambling products should have to follow**, in order to protect young people from developing harmful gambling behaviour?

Post one suggestion, then have a look at what other members of the Community are suggesting. Jump on to any threads you agree with and add what you can to the ideas until you think we, as a group, have a good list of what might stop young people from developing harmful patterns of gambling.

**Day 5**

**Exposure sites: advertisement edition**

Today we’re going to be talking more about advertising and all the ways that gambling might be promoted. These include ads for gambling brands or products, as well as promotional offers such as specials, bonuses and other inducements to gamble.

- **Where are young people most likely to come across gambling ads and promotional offers**?

Provide examples if you can!

*Select all that apply*: *Social Media; Web browsing; Gaming sites; Streaming sites; Semi-legal or illegal site (e.g. skin gambling sites, pirating, torrent); Phone apps (including gaming and non-gaming); Direct contact (including emails, texts, calls); TV/Radio; Print (e.g. magazines, newspapers, billboards, flyers, bus bench ads); Other (specify)*

- **Reach**

Are there any channels that gambling providers use that you think **best reach young people and get them to try gambling**?

In your answer, think about whether online, social media, television, sponsorship or any other types of promotion might get the attention of young people more than others, and why that is.

- **Message**

And what kind of**devices, gimmicks or messages** do you think gambling promoters use that **young people really take notice of**? 
In your answer, include what ads might say about gambling to get young people to try it and what gambling providers put in ads and promotional offers to get attention (and whether you think that works!) You might mention a memorable gambling ad or promotional offer and why it was memorable.

- **Eye catching ads: Apps**

The next couple of questions are about different places you might see gambling being promoted and what you think about how effective they are.

Have you seen any**gambling ads or promotional offers on any apps that you use on your mobile or tablet**? This might include playable ‘demos’ of games, forced ads or banners in mobile games etc.

Tell us about your experiences, and whether you think promoting gambling on apps could convince young people to gamble, and why.

- **Eye catching ads: Social Media**

Have you seen any **gambling ads or promotional offers on social media or when browsing online**?

Tell us about your experiences, what social media platforms are most likely to send you gambling ads. In your answer, include whether you think promoting gambling on social media and online could convince young people to gamble, and why.

- **Eye catching ads: Streaming**

Have you seen any **gambling ads or promotional offers on any content streaming sites**, including catch-up of live TV, twitch etc?

Tell us about your experiences, and in your answer, include whether you think promoting gambling on streaming sites could convince young people to gamble, and why.

- **Eye catching ads: Influencers**

Have you seen any**gambling ads or promotional offers where gambling was being promoted by any influencers or streamers** you follow?

Tell us about your experiences, and in your answer,**include whether you think influencers or streamers could convince young people to gamble**, and why.

- **Breaking down an ad**

Here’s an example of an ad that a few members of the Community have posted this week.

Let’s break it down!
Firstly, on the image below, **mark on the ad what you like and what you don't like**.  Use the comment box for each pin to tell us why you like or dislike parts of the ad, and add any other thoughts about pictures or people the ad uses.

Let's share some more thoughts about this particular ad.

1.         Outline what you think the key message of the ad is

2.         How convincing you think the ad is – do you think it works?

- **Advertiser Rules**

What kind of **rules should advertisers follow when it comes to gambling**?

Post your ideas, even if they are rules you think already exist. Read what other members are suggesting and **comment whether you agree with those rules** (politely and respectfully). Keep going until you think we, as a group, have a good list of rules and regulations for gambling ads.

- **Effectiveness of Advertiser Rules**

After you’ve read some other answers, add a comment below with your view on the following: **Do rules around advertising** (e.g. limiting where and times of day gambling providers can advertise) **actually benefit young people**? Can having these rules reduce the likelihood of young people developing harmful gambling behaviours?

If you’ve already included your thoughts on this in the last question, repeat your views here and really dive into WHY you think that.

**Day 6**

**Follow-up**

Today we’re going to ask you to answer questions and follow-ups your Moderator has left for you about your answers over the last couple of days. You can see these by clicking on the little 'mail' icon in the top right hand corner of your screen.

- **Harmful or harmless?**

Do you think that gambling is a harmful or harmless activity for young people?

Tell us why, and whether your opinion of this has changed as you got older.

Leave a comment on at least one other post from a Community member.

- **Types of harmful gambling**

What **types of gambling do you think are most harmful**? 
Please tell us why you think this.

- **Harmfulness of gambling**

What do you think “**harmful gambling**” might look like, and how would you know if someone has a problem with gambling? In your answer, let us know if there’s been any time in your life where gambling has been harmful to you, or resulted in a bad outcome.

*A ‘bad outcome’ might be a bad effect on your relationships, emotions, health, education, work, or finances.*

And has**anyone you know had a bad outcome from gambling**? Please describe the outcome, and what impact this had on you.

- **Harmfulness of gaming**

What do you think “**harmful gaming**” on **video and digital games** might look like, and how would you know if someone had a problem with playing these games? In your answer, let us know if there’s been any time in your life where gaming has been harmful to you, or resulted in a bad outcome.

A ‘bad outcome’ might be a bad effect on your relationships, emotions, health, education, work, or finances

- **Parental Rules**

What **kind of rules do your parents/guardians have for you about using the internet, gaming, and gambling**?

In your response, mention any rules about banned websites or games, permission to download or play certain games, how much money you could spend buying/downloading/playing gambling games, time limits, etc, and how this has changed as you've gotten older.

- **Family environment**

While growing up, were there other things about your**family environment** that may have**discouraged** you from gambling?

In your response, you might think about things like positive family relationships, family interests and activities that kept you busy, adults who may have guided and supported you to make good choices, and other aspects of your family life.

- **Peer environment**

While you have been growing up, were there things about your **friendships/peer environment** that may have **discouraged** you from gambling or made it less likely that you would gamble? In your response, you might think about things like influential friendships, interests and activities with your friends that kept you busy, how acceptable or not gambling was amongst your peers, any positive role models, and other aspects of your friendships.

- **Teach the children well**

What kind of **education and resources** should be provided to young people about gambling?

Post your ideas for the topics or information you think would be useful for young people, such as:

- ‘How to's
- Information on risk
- How this information might be shared, e.g. by parents/guardians, advertising run by the government etc

*Have a look at what other members of the Community are suggesting. Jump on to any threads you agree with and add what you can to the ideas until you think we, as a group, have a good list of what resources and education might stop young people from developing harmful patterns of gambling.*

*If you feel like you’ve learned anything by participating in this Community and talking to other people about their ideas, include it here! Do you think other young people would benefit from having these kinds of conversations?*

- **Reducing the Risk**

Together we’ve come up with a whole bunch of strategies and rules aimed at protecting young people from developing harmful patterns of gambling behaviour.

**What do you think would be the most important and effective strategy?**

Feel free to re-visit posts from the last couple of days to pick your favourite and tell us why you think it is the most important in protecting young people.

**Days 2 to 6**

- **Advertising**

Please**upload a screenshot or description of any gambling-related ads you’ve seen** yesterday, last night or earlier today.

*A reminder: Please DO NOT go looking for these adverts. We’re interested in what adverts you might see each day in your normal activities. If you don’t see any adverts on any day, that’s fine too. We’re really interested in ads you see online, such as in your social media feed, banners on websites, a promotion from a streamer you follow etc. But an ad you see on TV or elsewhere is fine too!*

Please post:

1. A screenshot of the ad (if you can). Make sure it doesn’t have your name or location in the pic!
2. A quick description, including where you saw it, what company it was for or what type of gambling it was for
3. What do you think the ad is trying to tell the people that see it?
4. Who do you think the ad is aimed at?
5. How does this ad make you feel about the brand or the gambling product?
6. How does this ad affect how excited you are to gamble in the future?

If you have not seen a gambling ad over the last day or so, write "did not see any ad" for your answer.

**Day 7**

**Procrastination Station**

**Reminder:**Sunday is the**last day**to catch up on anything the Moderators have sent you and finish up any tasks At the end of the day we'll be tallying everyone's participation and assigning gift vouchers according to participation level

- **Reflecting back**

We’re almost done! Before you answer the final questions, we’d like to ask about your experiences with the Community over the past week.

**Please respond with:**
1. Your favourite activity and why
2. Something you learned from being part of the Community
3. ANY feedback for us you think would make a Community like this easier, more interesting or more fun for young people!

- **Looking Ahead**

Do you have any plans to **start or increase your gambling when you turn 18**?
Tell us what you have in mind, and whether you feel like you know what to expect from gambling once you are an adult.

- **Any other comments**

Is there anything else about gambling and young people that you’d like to talk about that you haven’t mentioned before?
